# Various Free Flap Reconstruction Techniques after Hand and Foot Melanonychia Ablation: A Case Series

**DOI:** 10.3390/jcm13102811

**Published:** 2024-05-10

**Authors:** Seungjun Lee, Seokchan Eun

**Affiliations:** Department of Plastic and Reconstructive Surgery, Seoul National University Bundang Hospital, Seoul National University College of Medicine, Seoul 13620, Republic of Korea; winters88@naver.com

**Keywords:** melanonychia, free flap, melanoma, nail

## Abstract

(1) **Purpose**: The management of melanonychia is highly controversial. With growing melanonychia, in case of doubt, the entire lesion should be examined. It may appear similar to nail melanoma or may turn into melanoma. Here, we present surgical cases of nail bed total removal and free flap reconstruction. (2) **Methods**: Between 1 January 2020 and 31 December 2022, eleven patients were operated on for growing melanonychia, involving the hand and foot. After complete resection of the nail root and plate, immediate reconstruction was performed using a fasciocutaneous free flap. The authors describe the procedures in detail with a few illustrations and clinical photographs depicting the techniques. (3) **Results**: All patients underwent complete nail unit removal around the anatomic boundaries of the nail. Histology showed a nevus and no malignancy in all cases. We used three onychocutaneous flaps, three hypothenar flaps and five venous flaps. All flaps survived, with one case of partial necrosis which spontaneously healed with secondary intention. In the follow-up periods, there was no recurrence or nail regrowth. (4) **Conclusions**: These free flap techniques are very useful for total nail bed defect reconstruction after melanonychia lesion total ablation.

## 1. Introduction

Melanonychia is a pigmented lesion of the nail bed which is caused by increased melanocyte activity in the nail matrix and subsequent increased melanin deposition in the nail plate. Differential diagnosis from other diseases or tumors must be made [1]. Possible causes include nail melanoma, nevus, sunspots, racial and friction pigmentation, subungual hemorrhage, and bacterial or fungal infections. It is sometimes quite difficult to differentiate between melanonychia and subungal melanoma [2]. Late diagnosis of subungal melanoma prognosis is usually rather poor. Because of the morbidity associated with surgeries such as amputation, it is important to detect melanoma early and treat it aggressively. Clarifying the etiology of any melanonychia is quite important. However, histologic features of early melanoma remain a histologic challenge because they can be isolated. The literature discusses the management of melanonychia, but when in doubt, the surgeon should perform a gross examination of the lesion [2,3]. Also, the nail matrix biopsy procedure itself poses a high risk of postoperative split nail and other nail deformity. Even though a biopsy result at that time may be negative for malignancy, it does not foretell the upcoming future. It cannot guarantee the malignant transformation of current melanonychia and particularly in the expanding form. In this context, microsurgery can be used to reconstruct the nail unit after total nail plate resection. Here, we aimed to show the usefulness of the free flap technique to reconstruct the nail bed defect reconstruction in the hand and foot.

## 2. Materials and Methods

Between 1 January 2020 and 31 December 2022, eleven patients were operated on for growing melanonychia, involving the hand and foot. After complete resection of the nail bed and root, immediate reconstruction was performed using a fasciocutaneous free flap. The authors describe the various procedures in detail with a few illustrations and clinical photographs highlighting the techniques. A retrospective chart review involving eleven patients, from 1 January 2020 to 31 December 2022, was performed. The procedure included wide excision with nail plate ablation followed by microsurgical reconstruction. All 11 patients were treated by the highly experienced single microsurgeon (SC Eun). The color changes, extension processes and irregularities under the nail were criteria for complete removal of the nail bed, and one of the patients presented with periungual pigmentation (Hutchinson’s sign). All specimens were examined by the same pathologist. The following data were collected: age, sex, the affected digit/toe, flap type, clinical results with complications and recurrences. 

## 3. Surgical Technique

Before definite surgery, we obtained informed consent from the patient and their family after deep discussion. The entire surgery was performed under general anesthesia. After the complete nail plate and root avulsion, the defect was ready for flap reconstruction. For the onychocutaneous flap, the skin flap was elevated on the second toe dorsum. The digital artery, digital nerve and dorsal vein were identified and dissected in a proximal direction. The toe nail bed and small dorsal skin were included in the flap. The flap digital artery was anastomosed to the recipient’s proper digital artery. The flap subcutaneous vein located beneath the dermal layer was anastomosed to the dorsal subcutaneous vein of the recipient toe. The nerve was anastomosed to the appropriate palmar digital nerve. The donor defect could be full-thickness skin grafted from the inguinal area with easy uptake.

For the hypothenar flap, the exact location of the hypothenar perforator was determined using intravenous Incocyanine green dye intravenous injection under a fluoroscopic image. The ulnar border of the flap was first dissected through the skin, and a suprafascial dissection technique was used to identify the flap perforator. After flap harvest, the flap pedicle was anastomosed with the recipient vessels in an end-to-end manner, and the flap skin was sutured to the margin of the defect. The donor site was closed primarily.

For the venous free flap, we inflated the tourniquet to 300–350 mmHg to engorge the vein and mark it on the skin surface of the foot dorsum. After preparing the recipient site, one efferent vein and one afferent vein were included in this cutaneous flap. End-to-end anastomoses were performed under a microscope. The flap was inset without any tension, and the donor site defect was covered by a Limberg-style local flap using adjacent skin with no skin graft.

Postoperatively, the patients received lipo-prostaglandin E1 10 μg by continuous intravenous infusion for 6 days. Postoperative monitoring was performed using clinical examinations like flap color, refill and temperature. Doppler examination was not routinely performed because the flap size was very small, and clinical signs provided sufficient evidence for flap survival after successful vascular anastomosis. The hand was elevated to minimize postoperative swelling, and the operated finger was immobilized with a splint for 10 days. The patient was discharged after 7–10 days of admission, and we conducted follow-up care once every week for one month, then every two, three or six months follow-up for 5 years.

## 4. Results

Out of the 11 patients, 6 were men (54%), and 5 were women (46%). The mean age at time of diagnosis was 44.6 years (18–62 years). Six lesions (two thumbs) were located on the digits and five on the toes. Histopathologic findings revealed a junctional nevus or compound nevus in the nail bed for all the patients. The flaps used in these series include 3 onychocutaneous free flaps, 3 hypothenar free flaps and 5 venous free flaps. All the flaps survived well except one flap exhibited partial necrosis. All eleven patients achieved successful reconstruction with good-to-excellent functional and esthetic results (Table 1). 

## 5. Patient Reports

### 5.1. Patient 1

**Figure 1:**
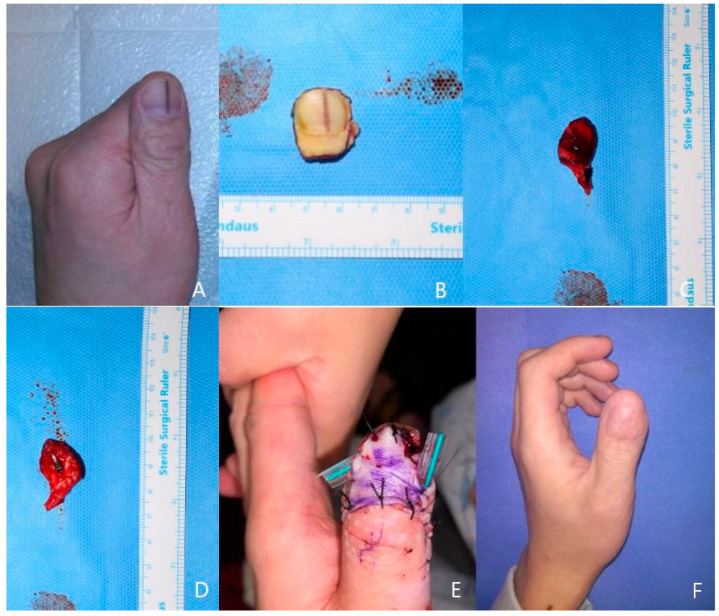
A case of 18-year-old male patient. (**A**) left thumb longitudinal melanonychia, (**B**) Totally excised melanonychia lesion, (**C**) harvested hypothenar flap, (**D**) Inner surface of flap(artery clamped), (**E**) Immediate postoperative photo of ipsilateral hypothenar free flap reconstrution. (**F**) post-operative 8-month photo.

A 21-year-old man visited the clinic with his left thumb melanonychia lesion which was found nine months ago. He was diagnosed with right eye retinoblastoma at age two and has been taking chemotherapy without surgery. His family wanted to totally remove the melanonychia lesion to prevent the malignant change, which was affected by the retinoblastoma medical history. The thumb radial-side digital artery and the dorsal cutaneous vein of the finger were prepared for anastomosis. After complete ablation of the nail bed and root area, we harvested the 1.8 × 2 cm-sized hypothenar free flap from the ipsilateral hand. The flap pedicle was anastomosed with the recipient vessel in an end-to-end manner, and the flap skin was sutured to the nail bed defect margin. The postoperative course was uneventful, and the patient regained the use of his fingers three weeks after surgery. The final pathology was a junctional nevus. The patient was satisfied with the functional and final esthetic results and with minimal donor site morbidity (Figure 1).

### 5.2. Patient 2

**Figure 2:**
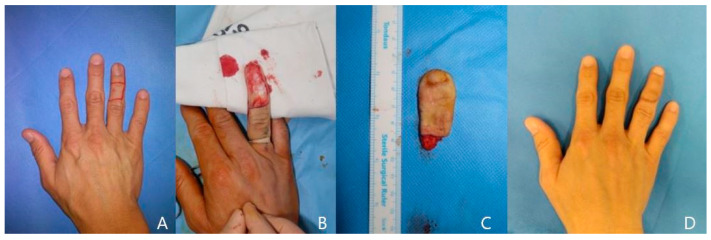
A case of a 50-year-old male patient. (**A**) Right 4th finger melanonychia with middle phalanx Bowen’s disease. (**B**) Defect after wide excision of nail bed and middle phalanx dorsal skin. (**C**) Long onychocutaneous free flap harvested from ipsilateral second finger. (**D**) Postoperative 6-month view.

A 50-year-old man was transferred from the dermatology section for right fourth finger Bowen’s disease. He had also had a melanonychia lesion on his nail bed for six months. He wanted to remove all of the premalignant lesion to remove the future potential of malignant change. We excised the entire nail bed and middle phalangeal skin in an en-bloc manner, and the radial-side digital artery and the dorsal cutaneous vein were exposed. We harvested the 2.5 × 5 cm-sized long onychocutaneous free flap from the right second toe. This flap was then transferred to the fingers, and the flap first dorsal metatarsal artery was anastomosed to the radial-side digital artery at the proximal phalanx level. A venous anastomosis was performed between each dorsal cutaneous vein. Finally, we sutured the flap-side digital nerves to the dorsal branch of the digital nerve. A relatively large secondary defect was closed with an adjacent great toe pulp pedicled flap. The postoperative course was uneventful, and final pathology was mild acanthosis and increased basal pigmentation. The transferred nail showed normal growth, and the finger showed excellent pinch and grasp motion (Figure 2).

### 5.3. Patient 3

**Figure 3:**
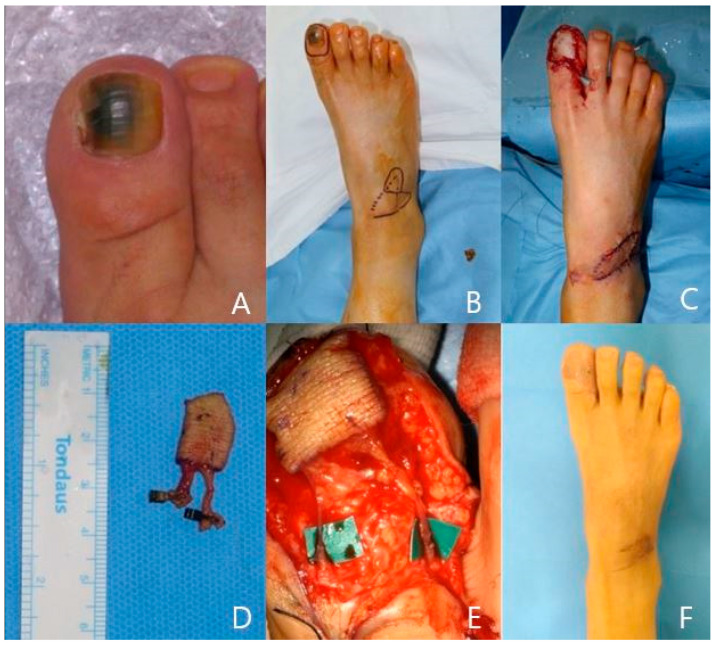
A case of a 47-year-old female patient. (**A**) Right great toe melanonychia preoperative photo. (**B**) Venous flap design on ipsilateral foot dorsum. (**C**) Flap transfer and donor site closure by local flap. (**D**) Harvested venous free flap with afferent and efferent veins. (**E**) Microanastomosis between digital artery and dorsal digital vein with two harvested veins. (**F**) Postoperative 11-month photo.

A 47-year-old woman has had a dark pigmented melanonychia lesion on her right great toe for three years. There was no malignancy-favoring evidence like signs of Hutchinson’s disease; however, she wanted to remove the whole nail bed. We planned to reconstruct the nail bed defect with a venous free flap from the ipsilateral foot dorsum. The 2 × 3 cm-sized flap had one afferent vein and one efferent vein. End-to-end anastomoses were performed with the toe ulnar-side digital artery and dorsal vein. The flap was inset without any tension, and the donor site defect was covered by a Limberg-style local flap, our novel technique using adjacent skin with no skin graft. The pathologic report was irregular acanthosis with melanocyte activation. The flap survived well, and the foot dorsum donor site shows minimal scar. The patient was satisfied with the final results (Figure 3).

### 5.4. Patient 4

**Figure 4:**
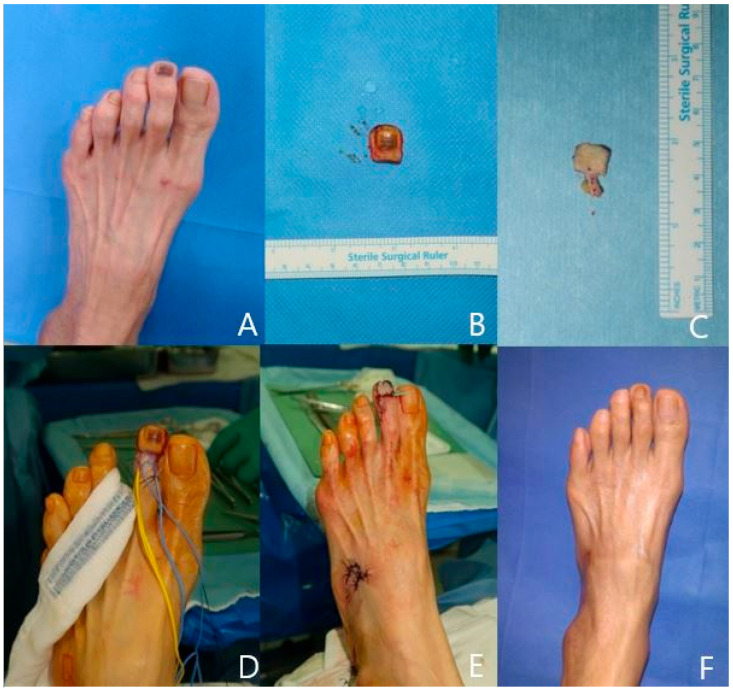
A case of a 51-year-old patient. (**A**) Preoperative photo. (**B**) Totally excised melanonychia lesion. (**C**) Small venous free flap harvested. (**D**) Recipient artery and vein prepared. (**E**) Immediately after flap transfer and donor site closure. (**F**) Postoperative 18-month photo.

A 51-year-old woman has had left second toe melanonychia for two years with no traumatic history. After discussion, we planned to remove the nail bed and reconstruct the nail bed defect with a venous free flap from the ipsilateral foot dorsum. One efferent vein and one afferent vein were included in the flap. End-to-end anastomoses were performed with an ulnar-side digital artery and dorsal vein. The donor site defect was covered by a Limberg-style local flap. The pathologic report was irregular acanthosis with melanocyte activation. The flap survived well, and the foot donor site shows very minimal scar (Figure 4). 

### 5.5. Patient 5

**Figure 5:**
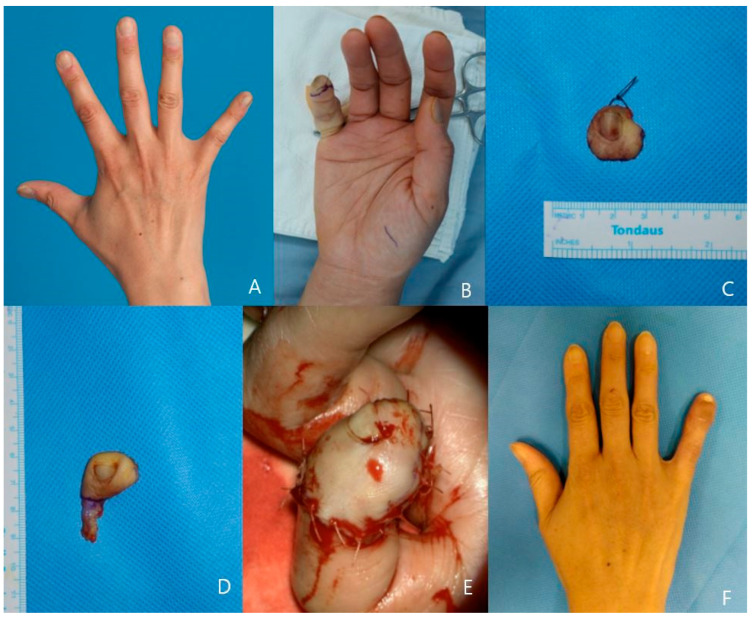
A case of a 46-year-old female patient. (**A**) Right 5th finger longitudinal melanonychia. (**B**) Hutchinson sign that crosses over the nail bed and extends to the fingertip skin. (**C**) Totally removed nail bed and surrounding skin. (**D**) Harvested onychocutaneous free flap with ipsilateral second toe. (**E**) Immediate postoperative view after microanastomosis with pin-point bleeding from the flap. (**F**) Postoperative 7-month photo.

A 46-year-old woman was transferred from another hospital for her right fifth finger melanonychia. She underwent a biopsy there two months ago, and her microscopic diagnosis for biopsy which was performed six months ago was junctional nevus with focal atypia and suggestive of dysplastic nevus. Her fingertip showed micro-Hutchinson signs with malignant potential. After deep discussion with the patient, we decided to remove the entire nail bed and surrounding skin widely. We excised the entire suspicious lesion in an en-bloc manner and harvested the 1.5 × 2 cm-sized onychocutaneous free flap from the right second toe. The flap first dorsal metatarsal artery was anastomosed to the radial-side digital artery at the middle phalanx level, and a venous anastomosis was performed between each dorsal cutaneous vein. Finally, we sutured the flap-side digital nerves to the dorsal branch of the digital nerve. The final pathology was acral melanoma with Clark level III (papillary dermis) and Breslow thickness 0.07 cm. The transplanted nail showed normal growth and showed no functional defect (Figure 5).

### 5.6. Patient 6

**Figure 6:**
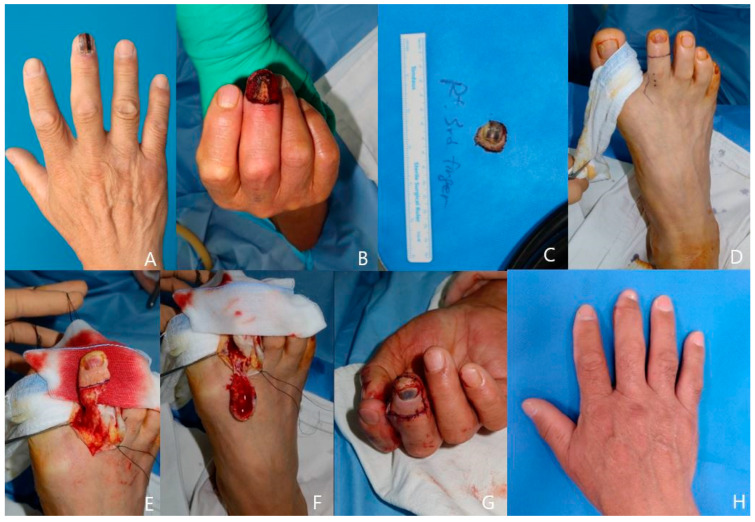
A case of a 65-year-old male patient. (**A**) Right 3rd finger melanonychia. (**B**) Defect after total ablation of nail bed and skin. (**C**) Removed lesion. (**D**) Onychocutaneous flap design on the ipsilateral second toe. (**E**) Flap elevation. (**F**) Inner side of harvested flap. (**G**) Immediate postoperative view after flap inset and microanastomosis. (**H**) Postoperative 9-month photo.

A 65-year-old man visited the clinic with his right third finger melanonychia lesion. The result of a punch biopsy performed at another department was atypical lentiginous melanocytic proliferation with cytologic atypia and mitosis of 1/whole specimen. It was highly suspicious of a malignant lesion. We checked the MRI, and bony cortex involvement was not observed. He and his family wanted to remove the entire lesion. After complete ablation of the nail bed and root area, we harvested the 1.5 × 2 cm-sized onychocutaneous free flap from the ipsilateral 2nd toe. The flap first dorsal metatarsal artery was anastomosed to the radial-side digital artery, and a venous anastomosis was established between the dorsal cutaneous vein. The patient was able to use his finger 4 weeks after the operation. The final pathology was acral melanoma (melanoma in situ) with Clark level I (epidermis) and Breslow thickness 0.05 cm. The patient expressed deep satisfaction with the final esthetic and functional results (Figure 6).

## 6. Discussion

The etiologies of melanonychia include lentigo, pigmented nevus, drug-induced hyperpigmentation, malnutrition-induced hyperpigmentation and subungual melanoma [1,4]. The diagnosis and management of melanonychia is still debated. On physical examination, physicians should search for the number, color, edge and width of bands, nodularity, and nail deformities in melanonychia [1,2]. Another approach for diagnosis is through dermatoscopy and biopsy. Dermoscopy is the most common diagnostic tool and less invasive than other procedures. Based on dermoscopic features, melanocytic nevus and subungal melanoma can be distinguished. Nail bed biopsy is a kind of invasive technique that many physicians hesitate to perform [1,3]. For the usual melanonychia patient, we do not apply the nail bed biopsy process. But, if Hutchinson’s sign (periungal pigmentation beyond the nail bed), the well-known clinical hallmark of subungual melanoma, or lines with irregular spacing and disruption of parallelism are found on dermoscopic examination, we perform punch biopsy to confirm subungal melanoma [2,3,4]. It is unestablished what percentage of melanonychia underwent malignant transformation. But, quite often, many melanoma patients had negative results at the initial biopsy. This is the reason why we remove the whole nail bed and root in a true melanonychia case. 

For nail bed defect reconstruction, various techniques can be used. Simple skin graft does not take well on the digital bone exposed bed. If ever taken, it has a low cosmetic appearance, suggesting the need for microsurgery [5]. Basically, an onychocutaneous flap can be harvested from the great toe or second toe if the patient agrees to it. The onchocutaneous flap in the short pedicle is our first choice any time because it has a better outlook and growing nail [6,7]. The flap digital artery is anastomosed to the recipient digital artery, and the toe dorsal vein is anastomosed to the toe dorsal vein. Even though the pedicle is short, there is no specific problem in vessel anastomosis. No large scars are left on the hands or feet [8,9]. This leads to cosmetic improvements and avoids prolonged surgical times.

If the patient wants simple skin coverage with no nail apparatus, we apply a hypothenar flap from the ipsilateral hand. This flap is based on multiple perforations arising from the palmar ulnar artery of the little finger side [10,11]. The hypothenar free flap has been chosen as an ideal option for small-to-medium-sized finger defect reconstruction because it has many advantages, including excellent color and texture match and excellent functional results [10]. Previous flap usage was for the volar surface and fingertip only, but we applied it first to the nail bed defect reconstruction [11,12]. Some disadvantages include tedious pedicle dissection and slight bulkiness of the flap. To avoid compression of the pedicle due to tight wound closure, skin grafting is sometimes necessary over the raw surface of the flap. In this case series, we made an effort to make it thin and fit for the finger dorsal surface defect. For a foot melanonychia case, we always harvest a venous free flap from the ipsilateral foot dorsum. After appropriate two-vein identification, flap dissection is very easy. At the proximal end of the flap, two skin veins are dissected and connected to the proper artery and dorsal vein of the recipient’s toes. A vein located near the recipient artery is selected as an afferent vein. Other flap donor sites like the wrist or forearm area provide an esthetically unpleasing scar. There exist venous valves and an arteriovenous shunt in the venous flap [13]. The afferent vein has reverse flow, which leads to some flap congestion. But marginal capillary ingrowth and flap skin thinness overcome this disadvantage. Some portions of the venous flap can take in the same manner as a full-thickness skin graft does [14,15].

This study has several limitations. This was a retrospective clinical study, not a randomized case–control study. A larger sample size is needed for further research. Additionally, it would be valuable to compare other cases of nail bed defects using different flaps or grafting methods.

## 7. Conclusions

For any doubtful melanonychia, relevant examination and well-planned surgery is necessary. Reconstruction of the nail excision can be performed using microsurgery. The three free flap techniques we used here have many advantages such as shorter operation time, low complication rate and satisfying esthetic outcomes.

## Figures and Tables

**Table 1:** Patient demographics and surgical details.

Patients	Age/Sex	Finger/Toe Affected	Combined Dx	Flap	Size	Complications /Recurrence
1	46/F	Rt 5th finger		Rt 2nd toe onychocutaneous free flap	1.5 × 2 cm	None
2	50/M	Rt 4th finger	Middle phalanx Bowen’s disease	Rt 2nd toe onychocutaneous free flap	2.0 × 5 cm	None
3	65/M	Rt 3rd finger		Lt 2nd toe onychocutaneous free flap	1.5 × 2 cm	None
4	62/M	Rt thumb		Rt hypothenar free flap	1.8 × 2 cm	None
5	18/M	Lt thumb	Rt eye retinoblastoma	Lt hypothenar free flap	1.8 × 2 cm	None
6	52/M	Lt 2nd finger		Lt hypothenar free flap	1.5 × 2 cm	None
7	47/F	Rt great toe	Thyroid cancer	Rt foot dorsum venous free flap	2 × 3 cm	None
8	31/F	Rt 2nd toe		Rt foot dorsum venous free flap	1 × 1 cm	Partial necrosis (20%)
9	48/M	Lt 4th toe		Lt foot dorsum venous free flap	1 × 1 cm	None
10	51/F	Lt 2nd toe		Lt foot dorsum venous free flap	1 × 1 cm	None
11	39/F	Rt 3rd toe		Rt foot dorsum venous free flap	1 × 1 cm	None

## Data Availability

The data presented in this study are available upon request from the corresponding author.
